# The helminth T2 RNase ω1 promotes metabolic homeostasis in an IL-33– and group 2 innate lymphoid cell–dependent mechanism

**DOI:** 10.1096/fj.15-277822

**Published:** 2015-10-21

**Authors:** Emily Hams, Rachel Bermingham, Felicity A. Wurlod, Andrew E. Hogan, Donal O’Shea, Roger J. Preston, Hans-Reimer Rodewald, Andrew N. J. McKenzie, Padraic G. Fallon

**Affiliations:** *Trinity Biomedical Sciences Institute, School of Medicine, Trinity College Dublin, Dublin, Ireland; ^†^National Children’s Research Centre, Our Lady’s Children’s Hospital, Dublin, Ireland; ^‡^Obesity Immunology, Education and Research Centre, St. Vincent’s University Hospital, University College Dublin, Dublin, Ireland; ^§^Department of Endocrinology, St. Vincent’s University Hospital, Elm Park, Dublin, Ireland; ^¶^Division of Cellular Immunology, German Cancer Research Center, Heidelberg, Germany; and ^‖^Medical Research Council Laboratory of Molecular Biology, Cambridge, United Kingdom

**Keywords:** obesity, adipocytes, inflammation

## Abstract

Induction of a type 2 cellular response in the white adipose tissue leads to weight loss and improves glucose homeostasis in obese animals. Injection of obese mice with recombinant helminth-derived *Schistosoma mansoni* egg-derived ω1 (ω1), a potent inducer of type 2 activation, improves metabolic status involving a mechanism reliant upon release of the type 2 initiator cytokine IL-33. IL-33 initiates the accumulation of group 2 innate lymphoid cells (ILC2s), eosinophils, and alternatively activated macrophages in the adipose tissue. IL-33 release from cells in the adipose tissue is mediated by the RNase activity of ω1; however, the ability of ω1 to improve metabolic status is reliant upon effective binding of ω1 to CD206. We demonstrate a novel mechanism for RNase-mediated release of IL-33 inducing ILC2-dependent improvements in the metabolic status of obese animals.— Hams, E., Bermingham, R., Wurlod, F. A., Hogan, A. E., O’Shea, D., Preston, R. J., Rodewald, H.-R., McKenzie, A. N. J., Fallon, P. G. The helminth T2 RNase ω1 promotes metabolic homeostasis in an IL-33– and group 2 innate lymphoid cell–dependent mechanism.

Obesity is associated with a low-grade proinflammatory state, with a central role for immune cells that infiltrate the adipose tissue contributing to localized and systemic inflammation and promoting insulin resistance and the development of metabolic syndrome (1, 2). The extent of obesity-related metabolic dysfunction directly correlates with the recruitment of proinflammatory cells, M1 classically activated macrophages (CAMs), and CD8^+^ T and CD4^+^ T helper (T_h_)-1 cells into the adipose tissue. Conversely, in lean individuals, the adipose tissue is populated with eosinophils, regulatory T (T_reg_) cells, NK T cells, group 2 innate lymphoid cells (ILC2s), and M2 alternatively activated macrophages (AAMs), which promote insulin sensitivity and metabolic homeostasis (3–5).

In experiments on obese mice, the artificial generation of a type 2 environment (*e.g.*, by infecting with type 2-inducing helminths such as *Nippostrongylus brasiliensis* and *Schistosoma mansoni*) has been shown to induce sustained weight loss and improvement in both glucose tolerance and insulin sensitivity (3, 4, 6). The helminth-mediated modulation was associated with a concomitant increase in eosinophil and ILC2 recruitment within the epididymal white adipose tissue (E-WAT) of the mice and the release of the type 2 cytokines IL-4, IL-5, and IL-13, resulting in increased AAMs (3, 4, 6). Furthermore, injection of obese mice with type 2-inducing *Schistosoma* egg-derived antigens also improved metabolic homeostasis (6). Analysis of soluble antigens in *S. mansoni* eggs identified the glycoprotein *S. mansoni* egg-derived ω1 (ω1), a T2 RNase (7), as the predominant type 2-inducing component (8, 9).

In this study, we sought to address the mechanisms underlying type 2 modulation of obesity and metabolic homeostasis using ω1 as a type 2-inducing molecule. Treatment of obese mice with ω1 induced a potent type 2 cellular response in the adipose tissue, with associated release of IL-33 from adipocytes, stimulating cells within the peritoneal cavity and adipose tissue to release type 2 cytokines, resulting in a localized accumulation of innate type 2 cells in the E-WAT. This capacity of ω1 to induce IL-33 release and modulate obesity is dependent upon the molecule’s RNase activity. Data presented herein demonstrate a potential RNase-mediated mechanism for modulation of type 2 immunity by an IL-33–dependent ILC2-mediated mechanism to alter obesity and stabilize glucose homeostasis.

## MATERIALS AND METHODS

### Animals

C57BL/6J, *Cd206*^−/−^, and *Rora^s^*^g/sg^ mice were purchased from The Jackson Laboratory (Bar Harbor, ME, USA). T1/ST2-deficient mice (*Il1rl1*^−/−^) (10), IL-33 citrine reporter mice (*Il33*^Cit/+^), and IL-33–deficient mice (*Il33*^Cit/Cit^) (11) were crossed to C57BL/6J in-house. Conditional retinoic acid orphan receptor (*Ror*)*a* floxed mice were generated, and *Rora*^fl/sg^ were crossed to *Il7r*^Cre^ (12), as previously described (13). Animals were housed in a specific pathogen-free facility in individually ventilated and filtered cages under positive pressure. All animal experiments were performed in compliance with the Irish Medicines Board regulations and approved by the Trinity College Dublin’s BioResources ethical review board.

### High-fat diet and *in vivo* metabolic testing

Age-matched mice were fed a high-fat diet (60% kcal fat, D12492; Research Diets, Inc., New Brunswick, NJ, USA) or control diet (10% kcal fat) for 8–16 wk as indicated (14). Glucose tolerance was assessed in mice unfed overnight and challenged with 2 g/kg glucose i.p. Insulin tolerance was tested in mice unfed for 4 h and challenged with 0.75 mU/g human insulin i.p. Blood glucose was measured at the times indicated using a glucometer (Abbott Laboratories, Abbott Park, IL, USA).

### *S. mansoni* egg antigen injection

*S. mansoni* eggs were isolated from the livers of infected mice and soluble egg antigens prepared as previously described (15). Native α1 and ω1 were isolated from soluble egg antigens by column chromatography, as previously described (16). Obese mice were injected intraperitoneally with 25 μg native α1 (d 0, 2, and 4) or 25 μg native ω1 (d 0, 2, and 4), and weight was monitored, as indicated in the figure legend (Supplemental Fig. S1).

### Recombinant ω1 production

Recombinant ω1, glycosylation mutant ω1 (N71/176Q; ω1^ΔGLY^), and RNase mutant ω1 (H58F; ω1Δ^RNase^) were expressed with a 6xHis tag in human embryonic kidney 293 cells, as previously described (8, 17). Recombinant ω1, ω1^ΔGLY^, and ω1Δ^RNase^ were purified from culture supernatants by nickel-affinity and gel-filtration chromatography. Purified protein was subject to detergent endotoxin extraction with ω1 preparations having <0.5 EU/mg protein (Lonza, Walkersville, MD, USA). The resultant proteins were checked for purity by SDS-PAGE. RNase activity was checked by incubating recombinant ω1 and ω1Δ^RNase^ (500 and 100 ng/ml, respectively) with 1 μg RNA isolated from murine bone marrow-derived macrophages for 1 h at 37°C. RNA integrity was determined by running on a 2% agarose gel and RNA visualized using ethidium bromide. Obese mice were injected with 25 μg ω1, ω1^ΔGLY^, or ω1Δ^RNase^ i.p. on d 0, 2, and 4 and weight monitored for 3 wk postinitial injection (Supplemental Fig. S2*B*). Corresponding groups of mice were treated with 25 μg endotoxin-free ovalbumin (OVA) as a glycoprotein control.

### Histology

E-WAT from mice was perfused and fixed in 10% formalin saline, and then paraffin embedded. Paraffin-embedded sections were cut to 4 μM and the slides stained with hematoxylin and eosin. For uncoupling protein 1 (UCP1) staining, slides were deparaffinized, and antigen retrieval was carried out by heating sections to 95°C in sodium citrate buffer. Sections were blocked with goat serum before incubation with rabbit anti-UCP1 antibody (ab10983; Abcam Inc., Cambridge, United Kingdom) diluted 1:100. Following peroxidase blocking, horseradish peroxidase-conjugated goat anti-rabbit (P0448; Dako, Glostrup, Denmark) was used as the secondary antibody, and sections were incubated at 1:1000 in PBS for 1 h at room temperature. For staining, diaminobenzidine chromagen (K3468; Dako) was used according to the manufacturer’s instructions, and Mayer’s hematoxylin was used to counterstain.

### Murine cell culture and stimulation

Murine adipocytes and macrophages were isolated from adipose tissue after incubation with 1 mg/ml collagenase D from *Clostridium histolyticum* (Roche Applied Science, Burgess Hill, United Kingdom). Briefly, the E-WAT was collected, mechanically shredded, and incubated with collagenase D at 37°C with gentle shaking for 1 h. After centrifugation at 400 *g*, the surface adipocyte fraction was gently collected and washed 3 times in PBS supplemented with 10% fetal calf serum. Adipocytes were identified as SSC^hi^FSC^hi^CD90^+^Sca-1^+^CD11b^−^ by flow cytometry. Macrophages (F4/80^+^ cells) were prepared from the stromal vascular fraction from the E-WAT. Adipocytes and macrophages were cultured in Roswell Park Memorial Institute medium (Buffalo, NY, USA) supplemented with 2 mM l-glutamine, 100 U/ml penicillin, and 100 μg/ml streptomycin at a density of 2 × 10^6^ cells/ml for 24 h, and the culture supernatant was discarded. Cells were incubated with 500 ng/ml recombinant ω1 for 1–24 h, as indicated in the figure legend (Fig. 4*E*). Culture supernatants were collected for cytokine profiling.

### Serum triglyceride quantification

Serum was assessed for triglyceride levels using the Abnova triglyceride quantification kit (Heidelberg, Germany) following the manufacturer’s instructions.

### Mouse cytokine ELISAs

Cytokine levels were quantified in tissue culture supernatants, peritoneal lavage fluid samples, and serum. Samples were analyzed by sandwich ELISAs to quantify levels of specific cytokines. IL-33 was measured with the DuoSet ELISA development system from R&D Systems (Abingdon, United Kingdom) following the manufacturer’s protocol.

### Human adipocyte isolation

Adipocytes were isolated from omental adipose tissue biopsies from obese patients (body mass index <50) undergoing elective bariatric surgery. Clinical studies were approved by the St. Vincent’s University Hospital, Dublin Ethics Committee. Written informed consent was obtained from each participant before commencement of research activities. Omental samples were processed to isolate adipocytes as described above. Adipocytes were incubated with 500 ng/ml ω1 for 3 and 24 h. IL-33 was quantified in the culture supernatant by ELISA (R&D Systems).

### Flow cytometry

E-WAT from lean and obese mice was mechanically shredded and incubated with 1 mg/ml collagenase D from *C. histolyticum* and a single-cell suspension prepared from the stromal vascular fraction (5). In addition, the adipocytes were collected and prepared for flow cytometry. Surface marker expression was assessed by flow cytometry with data collection on a CyAn (Beckman Coulter, High Wycombe, United Kingdom), and data were analyzed using FlowJo software (Treestar, Ashland, OR, USA). IL-33 expression was determined using Il33^Cit/+^ reporter mice. To identify ILC2s, cells were stained with BD Biosciences (Oxford, United Kingdom) mAbs CD8-APC (Ly-2), B220-APC (RA3-6B2), F4/80-APC (BM8), ICOS-PE (7E.17G9), and Siglec-F-APC (E50-2440); eBioscience Incorporated (Hatfield, United Kingdom) mAbs CD4-APC (RM4-5), CD11b-APC (M1/70), Gr-1-APC (RB6-8CS), and FcεR1-APC (MAR-1); and MD Biosciences (Zurich, Switzerland) mAb T1/ST2-FITC (DJ8). To identify eosinophils and AAMs, cells were stained with BD Biosciences mAbs Siglec-F-PE (E50-2440) and F4/80-APC (BM8), eBioscience Incorporated mAb CD11b-PerCP (M1/70), and BioLegend (London, United Kingdom) mAb CD206-PECy7 (C068C2). Prior to surface staining, all cells were incubated with Live/Dead Fixable Aqua stain (Molecular Probes, Invitrogen, Dublin, Ireland) to isolate dead cells. Using appropriate isotype controls, quadrants were drawn, and data were plotted on logarithmic scale density plots.

### RNA isolation and real-time PCR

RNA was isolated from homogenized E-WAT using the RNeasy kit and reverse transcribed using the QuantiTect Reverse Transcription Kit incorporating a genomic DNA elimination step (Qiagen, Germantown, MD, USA). Real-time quantitative PCR was performed on an ABI Prism 7900HT sequence detection system (Applied Biosystems, Dublin, Ireland) using predesigned TaqMan gene expression assays specific for murine UCP1 (Mm01244861_m1) and normalized to murine glyceraldehyde 3-phosphate dehydrogenase. Fold expression was calculated using the ΔΔ*C_t_* method of analysis.

### Statistics

Statistical analysis was performed using GraphPad InStat (GraphPad Software, La Jolla, CA, USA). Results are presented as means ± sem. Differences, indicated as 2-tailed *P* values, were considered significant when *P* > 0.05 as assessed by unpaired Student’s *t* test with Welch correction applied as necessary.

## RESULTS

### *S. mansoni* egg-associated proteins induce weight loss and promote glucose homeostasis in obese mice

Analysis of *S. mansoni* egg-excreted antigens has identified 2 major glycoproteins responsible for the immune-modulating activity of eggs: ω1 and *S. mansoni* egg-derived α1 [α1/IPSE (α1)] (7, 18). Although ω1 induces type 2 responses (8, 9, 17), α1/IPSE can induce basophils to produce IL-4, aiding a T_h_2 response (19). Intraperitoneal injection of obese mice with 25 μg native ω1, but not α1, induces a transient significant (*P* < 0.05) delay in weight gain (Supplemental Fig. S1*A*) and also significantly (*P* < 0.05) improved glucose tolerance (Supplemental Fig. S1*B*). These data indicate that when injected into obese mice, the helminth glycoprotein ω1, the major secreted T_h_2-inducing glycoprotein in *S. mansoni* eggs, but not α1/IPSE, can improve metabolic status.

**Figure 1.**
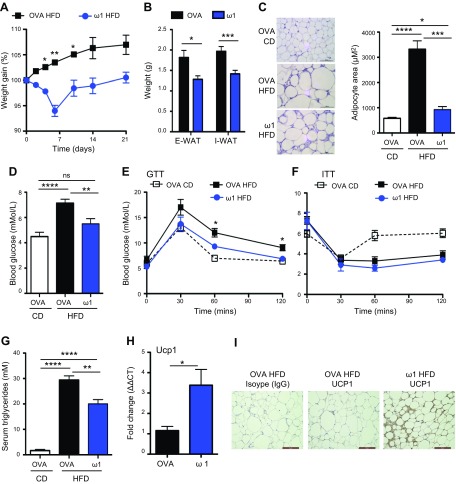
Recombinant ω1 induces weight loss and an improvement in glucose homeostasis in obese mice. *A*) Weight gain, expressed as a percentage from starting weight, in WT mice on high-fat diet (HFD) for 8 wk and treated with 25 μg recombinant ω1, or 25 μg OVA i.p. on d 0, 2, and 4. Weight was monitored for 21 d. *B*) Weight of excised E-WAT and inguinal white adipose tissue (I-WAT) in OVA- and ω1-treated mice at 6 d postinitial injection. *C*) Immunohistochemistry depicts hematoxylin and eosin staining of excised E-WAT from control diet (CD)-fed animals and HFD-fed animals treated with OVA or ω1. E-WAT was excised at d 6 postinitial injection. Adipocyte area was calculated from histologic slides. *D*–*F*) Blood glucose was assessed basally in unfed mice (*D*), and glucose tolerance was assessed after injection of 2 g/kg glucose i.p. at d 6 postinitial injection of ω1 (*E*); insulin tolerance was assessed in unfed mice after injection of 0.75 mU/g human insulin i.p. at d 6 postinitial injection of ω1 (*F*). *G*) Levels of triglyceride were determined in the serum of OVA- and ω1-treated mice. *H*, *I*) *Ucp1* expression (*H*) and UCP1+ cells (*I*) were determined in the E-WAT of OVA- and ω1-treated mice by quantitative PCR and immunohistochemistry, respectively. GTT, glucose tolerance test; ITT, insulin tolerance test. Data are representative of *n* = 6–8 ± sem from 3 independent experimental replicates. ns, not significant. *P < 0.05; **P < 0.01; ***P < 0.001; ****P < 0.0001. Scale bars, 50 μm (*C, I*).

To further address the immunologic mechanism underlying the ability of ω1 to modulate obesity, we generated recombinant ω1 from human embryonic kidney 293 cells (Supplemental Fig. S2*A*). A dose of 25 μg ω1 was administered by i.p. injection based on dose response and optimal delivery route experiments (Supplemental Fig. S2*C*, *D*). Treatment of obese mice with recombinant ω1 given intraperitoneally on d 0, 2, and 4 (Supplemental Fig. S2*B*) caused a rapid and significant (*P* < 0.01–0.05) weight loss relative to mice injected with OVA as a control glycoprotein, with peak weight loss on d 6 postinitial injection (**Fig. 1*A***). In addition, there was a significant (*P* < 0.01–0.001) decrease in adiposity, characterized by a decrease in the weight of both E-WAT and inguinal WAT in ω1-treated obese mice (Fig. 1*B*). This was also reflected in a significant (*P* < 0.01) reduction in adipocyte area of ω1-treated obese mice (Fig. 1*C*). Lean mice treated with ω1 had no alterations in weight gain, and additionally, food and water intake was comparable between ω1- or OVA-treated lean or obese mice (data not shown). Analysis of the effects of ω1 on metabolic parameters demonstrated that ω1 treatment significantly (*P* < 0.01) lowered fasting blood glucose in obese mice to a level comparable to mice fed a control diet (Fig. 1*D*), with significant (*P* < 0.05) decreases in glucose tolerance (Fig. 1*E*), although there was no significant improvement in insulin sensitivity (Fig. 1*F*). Consistent with the improvements in metabolic function, there was a decrease in serum triglyceride levels in obese mice treated with ω1 (Fig. 1*G*).

**Figure 2.**
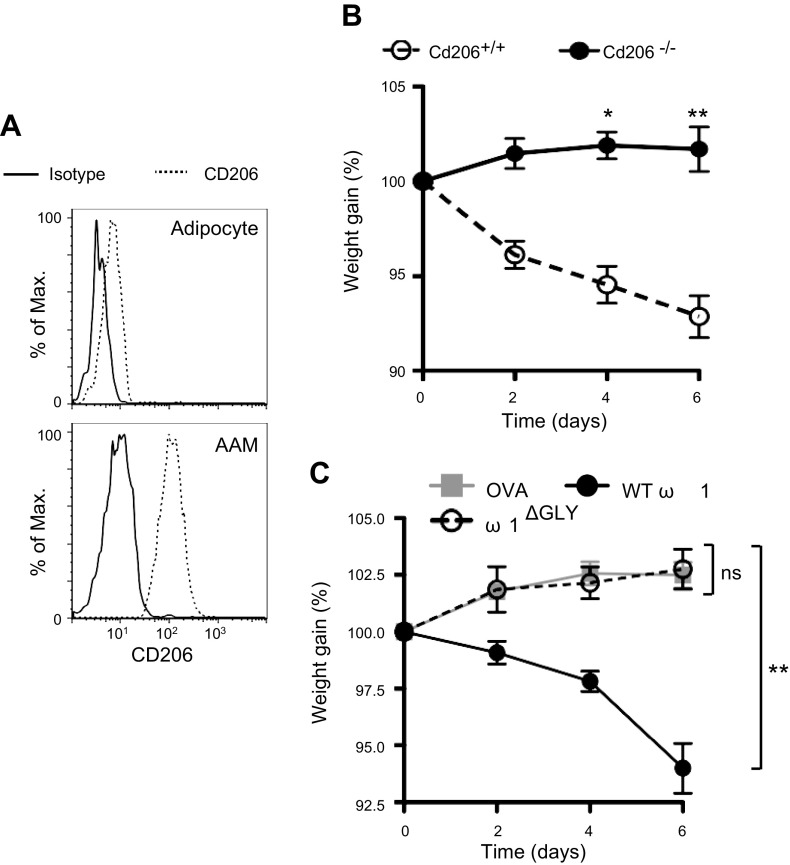
Expression of CD206 is required for the functional activity of ω1. *A*) CD206 expression on E-WAT adipocytes (SSC^hi^FSC^hi^CD90^+^Sca-1^+^CD11b^−^) and AAMs (CD11b^+^F4/80^hi^) was determined by flow cytometry. *B*) Weight gain, expressed as a percentage from starting weight, in WT and CD206-deficient mice on high-fat diet for 8 wk and treated with 25 μg ω1, or 25 μg OVA i.p. on d 0, 2, and 4. *C*) Weight gain, expressed as a percentage from starting weight, in WT mice on high-fat diet for 8 wk and treated with 25 μg ω1 (WT), 25 μg ω1^ΔGLY^, or 25 μg OVA i.p. on d 0, 2, and 4. Max., maximum. Data are representative of *n* = 2–6 ± sem from 2 independent experimental replicates. ns, not significant. *P < 0.05; **P < 0.01.

ω1 is a glycoprotein, and its action is partly mediated through its binding to the mannose receptor (CD206) on the cell surface (17). CD206 is expressed on both AAMs and adipocytes in the E-WAT (**Fig. 2*A***) of obese mice, suggesting that ω1 could act directly on such cells within the adipose tissue. Indeed, when injected into obese mice unable to signal *via* CD206 [*Cd206*^−/−^ (20)], ω1 did not cause weight loss (Fig. 2*B*). Furthermore, using recombinant ω1 with mutations in the sites responsible for glycosylation (N71/176Q; ω1^ΔGLY^), we show no effect on weight gain (Fig. 2*C*). These data indicate that binding of ω1 to CD206 is essential for its capacity to induce weight loss and alter the metabolic status of obese mice.

It has become apparent that the cellular composition of the adipose tissue is also partly responsible for the metabolic status of both mice and humans (21). Although WAT is an effective energy store, brown or beige adipocytes are responsible for regulating calorific expenditure, thus preventing weight gain in mice and humans (22, 23). The weight loss and metabolic changes in ω1-treated mice were associated with an increase in the expression of UCP1, a surrogate marker for beiging of WAT (24, 25), in the WAT (Fig. 1*I*). Therefore, ω1 improves metabolic function and induces beige cells within the adipose tissue.

### ω1 induces localized type 2 cellular responses in the E-WAT of obese mice

**Figure 3.**
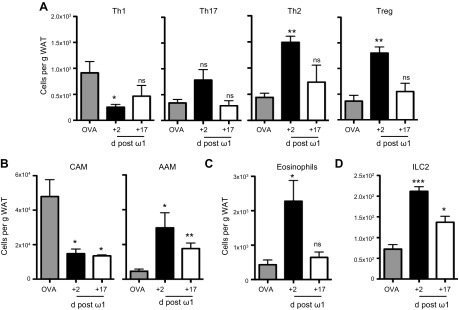
Recombinant ω1 induces a type 2 immune cell repertoire in the E-WAT of obese mice. Cellular infiltration into the E-WAT of obese mice treated with 25 μg ω1 or 25 μg OVA i.p. on d 0, 2, and 4 was assessed by flow cytometry 2 and 17 d postfinal injection of ω1. *A*) T_h_1 (CD4^+^IFN-γ^+^), T_h_2 (CD4^+^IL-4^+^), and T_h_17 (CD4^+^IL-17^+^) were assessed by intracellular cytokine staining, T_reg_ cells (CD4^+^FoxP3^+^) were determined by intranuclear staining. *B*) CAMs and AAMs were identified as CD11b^+^F4/80^+^CD206^lo^ and CD11b^+^F4/80^hi^CD206^hi^, respectively. *C*, *D*) Eosinophils (*C*) were identified as CD11b^+^SiglecF^+^ and ILC2s (*D*) as Lin^−^IL-7Rα^+^Sca-1^+^T1/ST2^+^KLRG1^+^. Data are representative of *n* = 6–8 ± sem from 3 independent experimental replicates. ns, not significant. *P < 0.05; **P < 0.01; ***P < 0.001.

Several studies have shown that the induction of a type 2 response, either systemically by infection (3, 6) or by injection of an exogenous cytokine (5), can induce alterations in the immune cell populations in the adipose tissue of obese mice leading to improved glucose homeostasis. Because ω1 is associated with the induction of IL-4 and IL-5 (8, 9, 17), we analyzed changes in the immune cell profile of the E-WAT in obese mice injected with ω1 at both 6 d and 21 d after initial treatment following intraperitoneal injections on d 0, 2, and 4 (d +2 and d +17, respectively). In obese mice injected with the control protein OVA, the cellularity of the E-WAT was proinflammatory, with T_h_1 cells and CAMs predominating (**Fig. 3*A*, *B***). In contrast, injection with ω1 dampened the obese proinflammatory cell signature with a significant (*P* < 0.05) decrease within the E-WAT of T_h_1 cells (Fig. 3*A*; Supplemental Fig. S3*A*) and CAMs (Fig. 3*B*; Supplemental Fig. S3*B*). Conversely, ω1 increased T_h_2 and T_reg_ cells (Fig. 3*A* and Supplemental Fig. S3*A*; *P* < 0.01), AAMs (Fig. 3*B*; Supplemental Fig. S3*B*; *P* < 0.05), eosinophils (Fig. 3*C*; Supplemental Fig. S3*C*; *P* < 0.05), and ILC2s (Fig. 3*D*; Supplemental Fig. S3*D*; *P* < 0.001) in the E-WAT of obese mice. These data demonstrate that the weight loss and downstream improvement in glucose metabolism in response to ω1 are associated with an increase in eosinophils and ILC2s in the WAT and with the induction of AAM polarization; these are cellular changes in adipose tissue known to impact on metabolic status (3, 26). Furthermore, these alterations in cellular composition can still be detected in the adipose tissue 17 d after treatment has ceased; although these cellular increases are no longer significant, these data do suggest that ω1 has a sustained beneficial effect on the cellular milieu in the E-WAT.

### ω1 induces the systemic and localized release of IL-33

**Figure 4.**
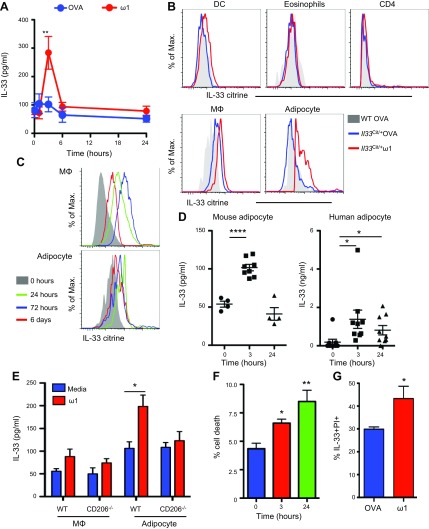
Recombinant ω1 induces the release of IL-33 from adipose tissue-associated cells *in vitro* and *in vivo*. *A*) Mice were injected intrapertitoneally with 25 μg OVA or ω1 and levels of IL-33 in the peritoneal lavage fluid determined at 1, 3, 6, and 24 h post ω1 interperitoneal injection. *B*) IL-33 expression by E-WAT DCs (CD11b^+^CD11c^+^F4/80^−^), AAMs (CD11b^+^F4/80^hi^CD206^hi^), eosinophils (CD11b^+^SiglecF^+^), CD4^+^ T cells, and adipocytes (SSC^hi^FSC^hi^CD90^+^Sca-1^+^CD11b^−^) 24 h after intraperitoneal treatment with 25 μg ω1 was determined using *Il33*^Cit/+^ reporter mice and displayed against WT mice treated with 25 μg endotoxin-free OVA. *C*) IL-33 expression (using *Il33*^Cit/+^ mice) from E-WAT macrophages and adipocytes 24 h, 72 h, and 6 d after treatment with 25 μg ω1. *D*) Mouse and human adipocytes were cultured *in vitro* in the presence of 500 ng/ml ω1 for 3 and 24 h; IL-33 levels were determined in the culture supernatant by ELISA. Each data point represents an individual subject (*n* = 4–12 mice per group; *n* = 10 patients). *E*) Adipocytes and macrophages isolated from the E-WAT from WT and *Cd206*^−/−^ mice were cultured with 500 ng/ml ω1 for 3 h; IL-33 levels were determined in the culture supernatant. *F*) Cell death (PI^+^ peritoneal exudate cells) was quantified in the peritoneal cavity 3 and 24 h after single treatment with ω1. *G*) IL-33^+^ adipocyte death (PI^+^) in the E-WAT expressed as a percentage, in response to treatment with ω1. Max., maximum. Data are representative of *n* = 3–6 ± sem from 2 independent experimental replicates. *P < 0.05; **P < 0.01; ****P < 0.0001.

Because ω1 induces potent type 2 responses, the role of the epithelial cell-derived type 2-promoting cytokines IL-33 and thymic stromal lymphopoietin was investigated. The ability of IL-25 to drive ILC2s and type 2 NK T cells, resulting in weight loss in obese mice, has been reported (5), and IL-33 is also involved in maintaining glucose homeostasis and promoting beiging of WAT (25, 27). In mice injected with ω1, whereas we do not observe significant induction of IL-25 or thymic stromal lymphopoietin, IL-33 is released into the peritoneal cavity 3 h postinjection (**Fig. 4*A***; data not shown). IL-33 is a member of the IL-1 superfamily, typically released by stromal cells, mast cells, and dendritic cells (DCs) (28). Using an IL-33 reporter mouse (11), we analyzed the cellular repertoire expressing IL-33 in the E-WAT. Although IL-33 expression was apparent in both macrophages and DCs to a small extent, the primary cellular source of IL-33 in the E-WAT was adipocyte (SSC^hi^FSC^hi^CD90^+^Sca-1^+^CD11b^−^) (Fig. 4*B*). Analysis of the kinetics of IL-33 expression demonstrates maximal expression of IL-33 in adipocytes 24 h after injection of ω1, whereas macrophages increase expression of IL-33 72 h after treatment (Fig. 4*C*). Furthermore, *in vitro* stimulation with ω1 induced significant (*P* < 0.0001) IL-33 release from adipocytes isolated from obese mice (Fig. 4*D*) and humans (Fig. 4*E*). Using CD206-deficient mice (*Cd206*^−/−^), we show that CD206 expression on the adipocyte is required for IL-33 release following *in vitro* treatment with ω1 (Fig. 4*D*). Interestingly, macrophages isolated from the E-WAT from either wild-type (WT) or *Cd206*^−/−^ mice do not release significant levels of IL-33 in response to *in vitro* culture with ω1; although expression of IL-33 is increased in these cells *in vivo* (Fig. 4*C*). The release of IL-33 from cells is often associated with cell death. In accordance with the cytotoxic nature of ω1, we observe increased death of peritoneal exudate cells in the peritoneal cavity following ω1 treatment (Fig. 4*E*). Analysis of the IL-33–expressing adipocytes demonstrated an increase in cell death associated with ω1 treatment (Fig. 4*F*). These data suggest that the localized cytotoxicity of ω1 induces E-WAT adipocytes to increase production and release of IL-33.

### RNase activity of ω1 induces weight loss and regulates glucose homeostasis

**Figure 5.**
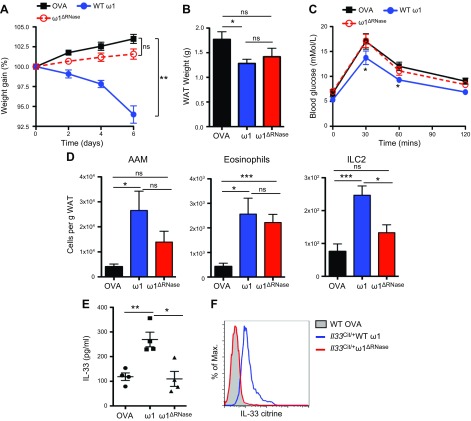
Weight loss and IL-33 induction by ω1 are mediated by RNase activity. *A*) Weight gain, expressed as a percentage from starting weight, in WT mice on high-fat diet for 8 wk and treated with 25 μg ω1 (WT), 25 μg ω1^ΔRNase^, or 25 μg OVA i.p. on d 0, 2, and 4. Weight was monitored for 6 d. *B*) Weight of E-WAT in OVA and WT and ω1^ΔRNase^-treated mice at 6 d postinitial injection. *C*) Glucose tolerance assessed after injection of 2 g/kg glucose i.p. at d 6 postinitial injection of WT or ω1^ΔRNase^. *D*) Cellular infiltration into the E-WAT was assessed by flow cytometry 6 d after initial injection of OVA, ω1, or ω1^ΔRNase^. AAMs were identified as CD11b^+^F4/80^hi^CD206^hi^, eosinophils were identified as CD11b^+^SiglecF^+^, and ILC2s as Lin^−^IL-7Rα^+^Sca-1^+^T1/ST2^+^KLRG1^+^. *E*) Mice were injected intraperitoneally with 25 μg ω1 or ω1^ΔRNase^, and peritoneal lavage fluid was collected after 6 h for ELISA analysis of IL-33. *F*) IL-33 expression in adipocytes (SSC^hi^FSC^hi^CD90^+^Sca-1^+^CD11b^−^Pref1^−^) was determined using *Il33*^Cit/+^ treated with 25 μg ω1 or ω1^ΔRNase^. Data are representative of *n* = 5–8 ± sem from 2 independent experimental replicates. ns, not significant. *P < 0.05; **P < 0.01; ***P < 0.001.

ω1 has been identified as a T2 RNase (7) (Supplemental Fig. S2*E*), a property shown to be integral to the ability of ω1 to induce IL-4 and IL-5 release (17). A recombinant ω1 RNase-null (ω1Δ^RNase^) mutant was generated, by substituting a phenylalanine residue in the RNase catalytic domain with a histidine residue (H58F), that was devoid of RNase activity (Supplemental Fig. S2*E*). Treating obese mice with ω1Δ^RNase^ did not induce significant weight loss or a significant reduction in adiposity (**Fig. 5*A*, *B***). Furthermore, ω1Δ^RNase^ did not improve glucose tolerance in obese mice (Fig. 5*C*). The absence of functional RNase activity also decreased type 2 cell infiltration into the E-WAT, with fewer ILC2s and AAMs observed; although interestingly, eosinophil infiltration was still significantly (*P* < 0.001) increased (Fig. 5*D*). In contrast to the mutant ω1Δ^RNase^ protein, recombinant ω1 with RNase activity (Supplemental Fig. S2*E*) was efficacious in modulating these parameters in obese mice (Fig. 5*A*–*D*). Furthermore, RNase activity was required for ω1-elicited release of IL-33 into the peritoneal cavity of mice (Fig. 5*E*) as well as inducing IL-33 expression in adipocytes (Fig. 5*F*). These data confirm a role for the RNase activity of ω1 in the induction of metabolic changes and also demonstrate an RNase-mediated mechanism for the stimulation of the release of IL-33.

### Weight loss and type 2 cell induction by ω1 are dependent on IL-33

**Figure 6.**
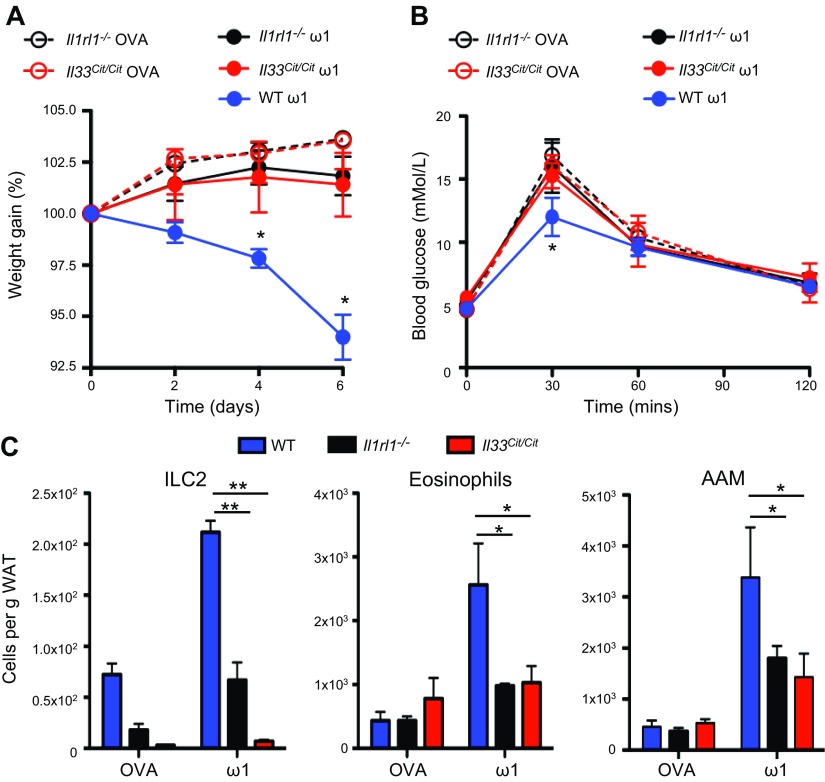
Recombinant ω1 does not induce significant weight loss in the absence of IL-33 or IL-33–mediated signaling. *A*) Weight gain, expressed as a percentage from starting weight, in WT mice, IL-33R–deficient mice (*Il1rl1*^−/−^), and IL-33–deficient mice (*Il33*^Cit/Cit^) treated with 25 μg ω1 or 25 μg OVA i.p. on d 0, 2, and 4. *B*) Blood glucose tolerance was determined at d 6 postinitial injection of ω1. *C*) The presence of ILC2s (Lin^−^IL-7Rα^+^Sca-1^+^T1/ST2^+^KLRG1^+^), eosinophils (CD11b^+^SiglecF^+^), and AAMs (CD11b^+^F4/80^hi^CD206^hi^) in the E-WAT of WT, *Il1rl1*^−/−^, and *Il33*^Cit/Cit^ was determined by flow cytometry. Data are representative of *n* = 3–6 ± sem from 2 independent experimental replicates. *P < 0.05; **P < 0.01.

To formally validate a role for IL-33 in ω1-mediated weight loss, obese IL-33 receptor (IL-33R; T1/ST2)–deficient (*Il1rl1*^−/−^) and IL-33–deficient (*Il33*^Cit/Cit^) mice were injected with ω1. In the absence of IL-33 and IL-33–mediated signaling, ω1 did not induce significant weight loss in obese animals as compared to WT animals (**Fig. 6*A***). Furthermore, there was no alteration in glucose tolerance in IL-33 pathway-deficient obese mice treated with ω1 (Fig. 6*B*). In support of ω1-induced weight loss and the changing cellularity of E-WAT being IL-33 dependent, injection of ω1 into obese IL-33– and IL-33R–deficient mice did not significantly induce ILC2s, eosinophils, or AAMs in the E-WAT (Fig. 6*C*).

**Figure 7.**
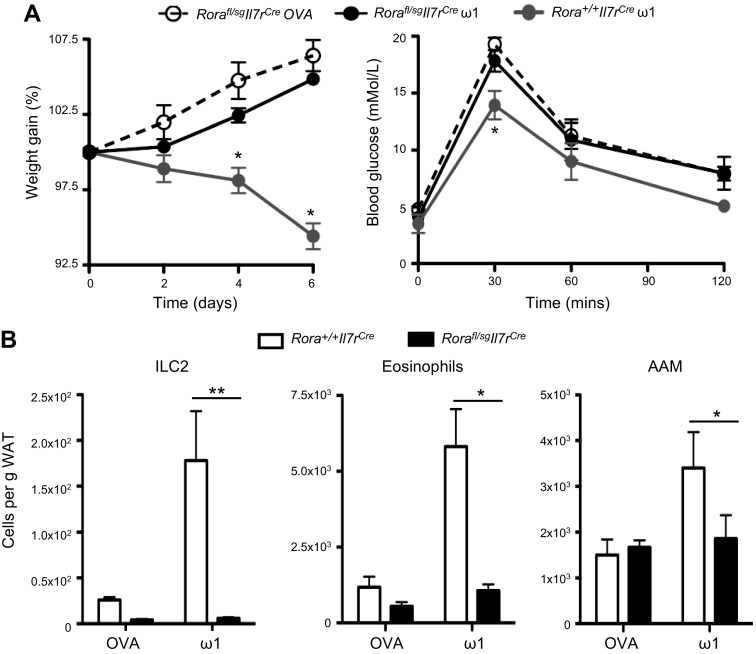
The activity of ω1 is ILC2 dependent. *A*) Weight gain and blood glucose tolerance were determined in ILC2-deficient *Rora*^fl/sg^*Il7r*^Cre^ and control *Rora*^fl/+^*Il7r*^Cre^ mice treated with 25 μg ω1 or 25 μg OVA i.p. on d 0, 2, and 4. *B*) The presence of ILC2s (Lin^−^IL-7Rα^+^Sca-1^+^T1/ST2^+^KLRG1^+^), eosinophils (CD11b^+^SiglecF^+^), and AAMs (CD11b^+^F4/80^hi^CD206^hi^) in the E-WAT of *Rora*^fl/sg^*Il7r*^Cre^ and *Rora*^fl/+^*Il7r*^Cre^ mice was determined by flow cytometry. Data are representative of *n* = 3–6 ± sem from 2 independent experimental replicates. *P < 0.05; **P < 0.01.

We next investigated the identity of the IL-33–responsive cells responsible for weight loss in the ω1-treated mice. IL-33 is a potent inducer of ILC2s in mice, and these cells have been identified recently as playing important roles in the activation and recruitment of eosinophils and AAMs in the E-WAT (4, 25). ILC2s are reliant upon the transcription factor RORα for their development, and staggerer mice (*Rorα*^sg/sg^), which have a natural mutation in the gene encoding RORα, are specifically deficient in ILC2s (29). To address the role of ILC2s in ω1-induced weight loss, ILC2-deficient *Rorα*^fl/sg^*Il7r*^Cre^ mice were used (13). These mice demonstrate a complete ablation of ILC2s (**Fig. 7*A***), in the context of this study we do not see significant reductions in the basal levels in any other cell population, however, the depletion of ILC2s can potentially impact CD4^+^ T cells, however, we do not observe any associated decrease in CD4+ T cell populations in the E-WAT [(13); data not shown]. Strikingly, administration of ω1 to obese *Rora*^fl/sg^*Il7r*^Cre^ mice demonstrated that ablation of ILC2s prevented significant weight loss, in contrast to the sustained weight loss in ω1-treated WT control *Rora*^fl/+^*Il7r*^Cre^ mice (Fig. 7*A*). Furthermore, the ability of ω1 to improve glucose tolerance was ablated in mice deficient in ILC2s (Fig. 7*A*). In addition, in the absence of ILC2s, injection of obese mice with ω1 failed to induce infiltration of eosinophils and AAMs into the E-WAT of obese mice (Fig. 7*B*). These data indicate that ω1 requires IL-33 and ILC2s to cause the alteration in the immune cell milieu in the E-WAT, resulting in increased eosinophilia and localized AAM polarization, which are associated with an improvement in metabolic parameters.

## DISCUSSION

Obesity and metabolic disorders are rapidly becoming an epidemic and a major cause of morbidity in the developed world. Many studies have focused on the immunologic causes of obesity and have associated the obese state with a low-grade inflammatory response in the adipose tissue (30). By promoting a more type 2 anti-inflammatory environment both systemically and in the adipose tissue, weight loss and an improvement in metabolic status can be achieved (3–5, 24, 25). Data presented herein identify a role for an isolated helminth egg-derived T2 RNase, ω1, to improve metabolic homeostasis in obese animals by a mechanism dependent on driving IL-33 release and localized ILC2 recruitment.

Studies have previously used helminths as a method of inducing a systemic type 2 response and have demonstrated favorable effects on metabolic parameters in mice (3, 6). This return to metabolic homeostasis in helminth-infected obese mice is associated with increased eosinophils, ILC2s, and AAMs in the adipose tissue, a change in immune status that can be maintained through chronic infection (3, 6). This study provides mechanistic insight into helminth modulation of obesity and identifies a specific protein released by helminth eggs, which is capable of driving a similar alteration in immune and metabolic parameters as that observed in live helminth infection. Studies have focused on the ability of ω1 to drive DCs to polarize naive CD4 cells to a T_h_2 phenotype, *via* a mechanism requiring both glycosylation and RNase activity (17). Although glycosylation is required for internalization of ω1 *via* binding to CD206, the RNase interferes with protein synthesis by global cleavage of rRNA and mRNA once translocated to the cytosol, enabling ω1 to condition DCs for priming of T_h_2 responses (17). The ω1 RNase is not unique in its association with induction of a T_h_2 response; the birch pollen antigen Bet v-1 has been identified as an RNase (31), and the fungal RNase Aspf-1 has allergenic capacity (32). These studies suggest that the ability to induce a T_h_2 response *via* RNase activity may not be unique to helminths.

The mechanisms described in this study identify a central role for IL-33 in obesity and metabolic homeostasis. Indeed, IL-33 is rapidly becoming accepted as an important factor in limiting obesity and metabolic dysregulation with several groups identifying a central role for IL-33 in metabolism (25, 27, 33). We have shown that ω1 is able to drive IL-33 release from cells, in particular adipocytes, through its intrinsic RNase activity. This ω1-mediated release of IL-33 not only alters the immune environment within the adipose tissue but also promotes the beiging of WAT, in a mechanism that limits adiposity by increasing calorific expenditure (24). Furthermore, we have shown that, in addition to immune cells within the WAT, adipocytes themselves are a target for ω1 and express and release IL-33. This appears to be mediated *via* the RNase function of the protein because IL-33 expression is ablated if the RNase function of ω1 is mutated. However, ω1 is a hepatotoxin (18, 34), and IL-33 release is often associated with cellular necrosis (35). Indeed, we do observe localized cell death *in vitro* in response to ω1 treatment, although at low levels. Furthermore, <50% of the IL-33–expressing adipocytes are dying; therefore, whereas cell death is likely to be a mechanism inducing IL-33 and thus causing weight loss, there is potentially another mechanism underlying IL-33 release in response to ω1.

**Figure 8.**
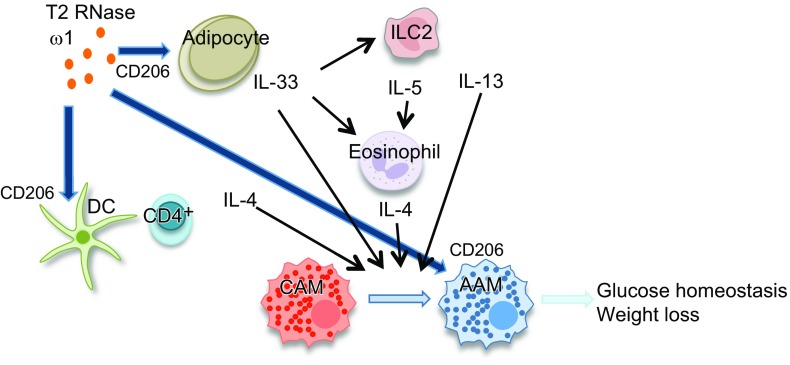
Schematic of the actions of ω1 in the adipose tissue. ω1 acting *via* CD206 on the surface of DCs and adipocytes can drive the production of type 2 cytokines, resulting in the downstream polarization of CAMs in the adipose tissue of obese mice, to an AAM phenotype, resulting in downstream stabilization of glucose homeostasis.

IL-33 is an important inducer of ILC2, a cell widely implicated in improving metabolic status in obese mice (4, 5, 25). Notably, we also observed that ω1-induced ILC2s were required for weight loss and improving glucose tolerance. However, due to the lack of CD206 on the surface of ILC2s (36), it is unlikely that ω1 acts directly on ILC2s. Instead, we propose a mechanism where ω1 binding to CD206 on adipocytes initially induces IL-33 release in part *via* an RNase-mediated mechanism, thereby inducing ILC2s and other innate cells to release IL-4, IL-5, and IL-13 resulting in a switch in polarization of macrophages to AAMs (**Fig. 8**).

This study provides further mechanistic insight into the immune-mediated regulation of obesity. We demonstrate an important role for RNase-mediated IL-33 release in promoting metabolic homeostasis in obese animals. We also identify the ability of ω1 to induce IL-33 release from human adipocytes, suggesting that any beneficial effect that ω1 treatment has in mice has the additional potential to be beneficial in humans. Data presented herein not only provide novel mechanistic insights into the roles of IL-33 and ILC2s in promoting metabolic homeostasis, but they also raise the potential beneficial therapeutic use of type 2-inducing RNases in the treatment and management of obesity and obesity-related metabolic disorders.

## Supplementary Material

Supplemental Data

## Abbreviations

α1/IPSE: *Schistosoma mansoni* egg-derived α1
ω1: *Schistosoma mansoni* egg-derived ω1
AAM: alternatively activated macrophage
CAM: classically activated macrophage
DC: dendritic cell
E-WAT: epididymal white adipose tissue
IL-33R: IL-33 receptor
ILC2: group 2 innate lymphoid cell
OVA: ovalbumin
ROR: retinoic acid orphan receptor
T_h_: T helper
T_reg_: regulatory T
UCP1: uncoupling protein 1
WAT: white adipose tissue
WT: wild-type
