# Supplementary material for: The helminth T2 RNase ω1 promotes metabolic homeostasis in an IL-33– and group 2 innate lymphoid cell–dependent mechanism
Source: FASEB J. 2015 Oct 21;30(2):824–35. doi: 10.1096/fj.15-277822 (PMC4973506; doi:10.1096/fj.15-277822)
Supplement: Supplemental Data [file supp_30_2_824__index.html]

The helminth T2 RNase ω1 promotes metabolic homeostasis in an IL-33 and group 2 innate lymphoid cell-dependent mechanism — The helminth T2 RNase ω1 promotes metabolic homeostasis in an IL-33– and group 2 innate lymphoid cell–dependent mechanism — Supplemental Data 

# The helminth T2 RNase ω1 promotes metabolic homeostasis in an IL-33– and group 2 innate lymphoid cell–dependent mechanism

## Supplemental Data

- Supplemental Data
- Supplemental Data
- Supplemental Data
