# Supplementary material for: The helminth T2 RNase ω1 promotes metabolic homeostasis in an IL-33– and group 2 innate lymphoid cell–dependent mechanism
Source: FASEB J. 2015 Oct 21;30(2):824–35. doi: 10.1096/fj.15-277822 (PMC4973506; doi:10.1096/fj.15-277822)
Supplement: Supplemental Data [file supp_fj.15-277822_Supplemental_Figure1.pdf]

Figure S1

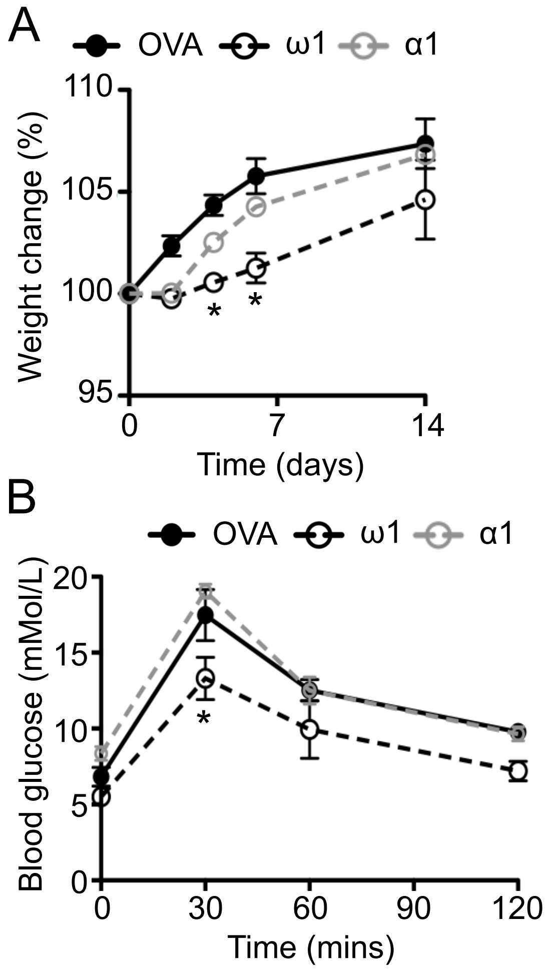

Figure S1: Native ω1, but not α1, induces transient weight loss and an improvement in glucose homeostasis. (**A**) Weight gain, expressed as a percentage from starting weight, in WT mice on high fat diet (HFD) for 8 weeks, and treated with 25 μg i.p. native α1, ω1, or 25 μg OVA i.p. on days 0, 2 and 4. Weight was monitored for 14 days. (**B**) Glucose tolerance was assessed after injection of 2 g/kg glucose i.p. at day 6 post initial injection of α1 or ω1. Data are representative of n=3-5 (+/- SEM) from 2 independent experimental replicates (*P<0.05).
