# Supplementary material for: The helminth T2 RNase ω1 promotes metabolic homeostasis in an IL-33– and group 2 innate lymphoid cell–dependent mechanism
Source: FASEB J. 2015 Oct 21;30(2):824–35. doi: 10.1096/fj.15-277822 (PMC4973506; doi:10.1096/fj.15-277822)
Supplement: Supplemental Data [file supp_fj.15-277822_Supplemental_Figure2.pdf]

Figure S2

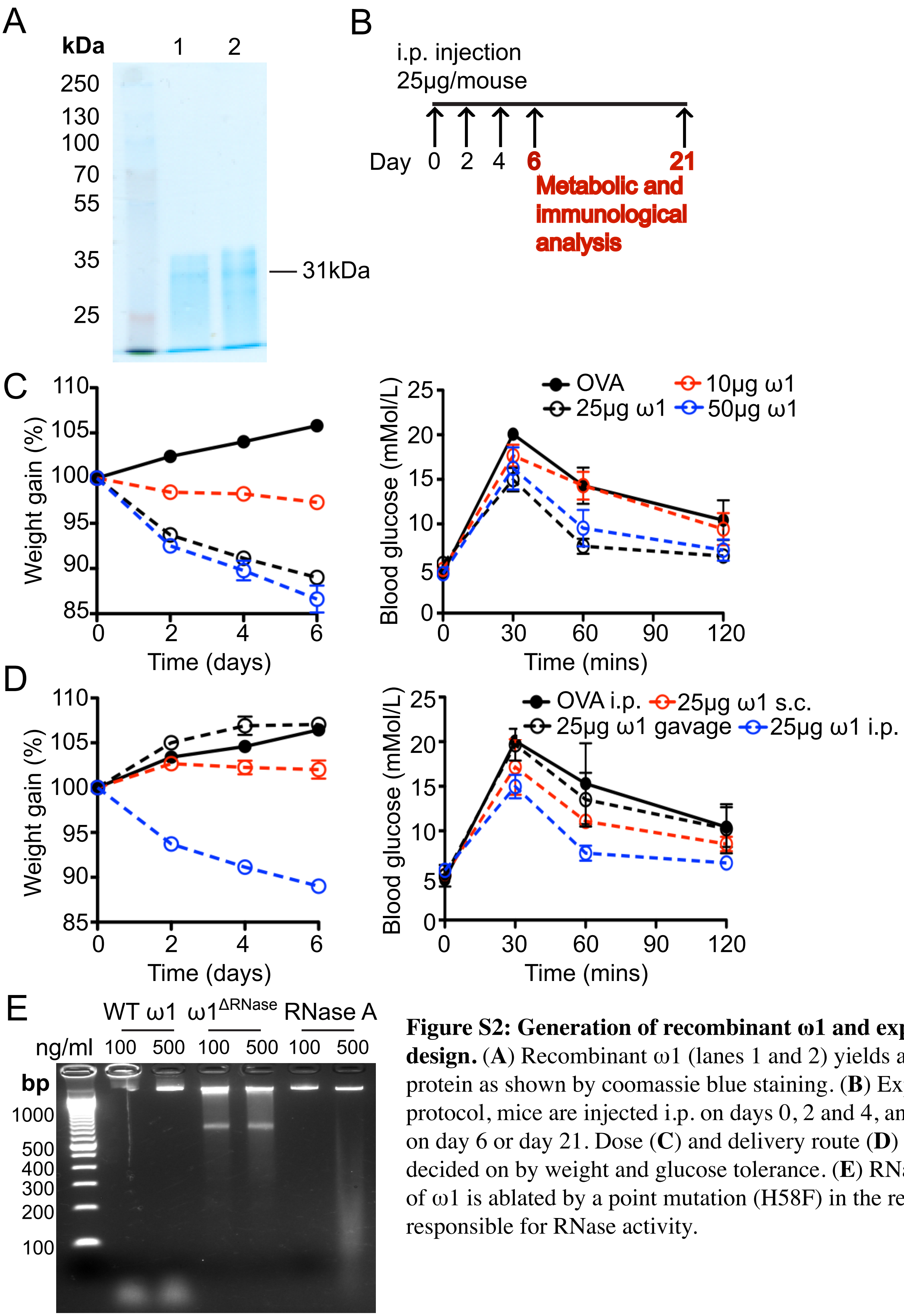

Figure S2: Generation of recombinant ω1 and experimental design. (**A**) Recombinant ω1 (lanes 1 and 2) yields a 31kDa protein as shown by coomassie blue staining. (**B**) Experimental protocol, mice are injected i.p. on days 0, 2 and 4, and sacrificed on day 6 or day 21. Dose (**C**) and delivery route (**D**) optimisation, decided on by weight and glucose tolerance. (**E**) RNase function of ω1 is ablated by a point mutation (H58F) in the region responsible for RNase activity.
