# Supplementary material for: The helminth T2 RNase ω1 promotes metabolic homeostasis in an IL-33– and group 2 innate lymphoid cell–dependent mechanism
Source: FASEB J. 2015 Oct 21;30(2):824–35. doi: 10.1096/fj.15-277822 (PMC4973506; doi:10.1096/fj.15-277822)
Supplement: Supplemental Data [file supp_fj.15-277822_Supplemental_Figure3.pdf]

Figure S3

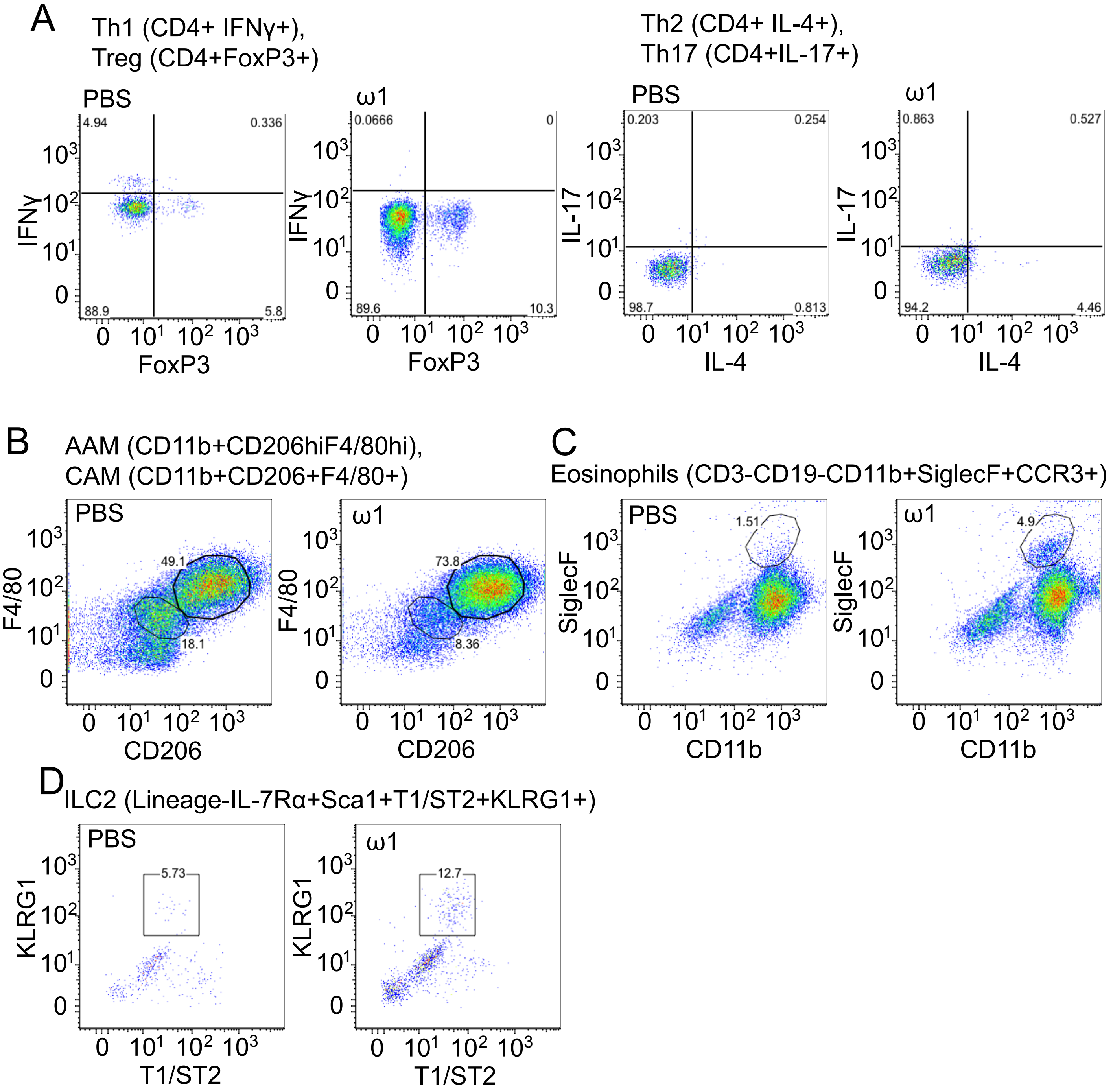

Figure S3: Representative gating strategies for E-WAT flow cytometry. Th1 (CD4+IFN-γ+), Th2 (CD4+IL-4+) and Th17 (CD4+IL-17+) were assessed by intracellular cytokine staining, Tregs (CD4+FoxP3+) were determined by intranuclear staining (**A**). Classically (CAM) and alternatively activated macrophages (AAM) were identified as CD11b+F4/80+CD206lo and CD11b+F4/80hiCD206hi respectively (**B**). Eosinophils were identified as CD11b+SiglecF+ (**C**), and ILC2 as Lineage-IL-7Rα+Sca-1+T1/ST2+KLRG1+ (**D**). Data are representative of n=6 (+/- SEM) from 3 independent experimental replicates.
